# The complete genomic sequence of *Sugarcane mosaic virus* from *Canna* spp. in China

**DOI:** 10.1186/s12985-018-1058-8

**Published:** 2018-09-24

**Authors:** W Tang, Z-Y Yan, T-S Zhu, X-J Xu, X -D Li, Y-P Tian

**Affiliations:** 1 Shandong Province Key Laboratory of Agricultural Microbiology, Department of Plant Pathology, College of Plant Protection, Shandong Agricultural University, Tai’an, Shandong 271018 People’s Republic of China; 2 Shandong Provincial Station for Plant Protection, Ji’nan, Shandong 250100 People’s Republic of China; 3 College of Plant Science and Technology, Tarim University, Alar, 843300 Xinjiang China

**Keywords:** *Canna* spp., Complete genome, Phylogenetic analysis, SCMV

## Abstract

**Background:**

*Sugarcane mosaic virus* (SCMV) is the prevalent virus inducing maize dwarf mosaic and sugarcane mosaic diseases in China. According to the phylogenetic results of the complete genomic and coat protein gene sequences, SCMV was divided into four or five molecular groups, respectively. Previously, we detected SCMV isolates of group SO from *Canna* spp*.* in Ji’nan, Shandong province, China.

**Findings:**

In this study, we collected two SCMV isolates infecting *Canna* spp. in Ji’nan (Canna-Ji’nan) and Tai’an (Canna-Tai’an) of Shandong, China. Their complete genome sequences had genome of 9576 nucleotides and contained a large open reading frame encoding a polyprotein of 3063 amino acids. The phylogenetic analysis showed that the both Canna-Ji’nan and Canna-Tai’an were clustered into an independent group based on the complete genome sequence.

**Conclusion:**

In this study, we report the complete genome sequences of SCMV infecting *Canna* spp. from Ji’nan and Tai’an. This is the first report on SCMV belonging to SO group.

**Electronic supplementary material:**

The online version of this article (10.1186/s12985-018-1058-8) contains supplementary material, which is available to authorized users.

## Body of text

*Sugarcane mosaic virus* (SCMV) is a species of the genus *Potyvirus* in the *Potyviridae* family and mainly infects plants of monocotyledons including maize, sorghum, sugarcane and canna. It is the major pathogen of sugarcane mosaic disease and maize dwarf mosaic disease and causes significant yield losses in China [1, 2]. SCMV has flexuous filamental particles of 700~ 750 nm long and can be transmitted by aphids in a non-persistent manner. The SCMV genome is single-stranded, positive RNA containing a single open reading frame (ORF), which code a large polyprotein and a truncated frame-shift product [3]. SCMV isolates can be clustered to four groups according to the phylogenetic analysis of the complete genomic sequences [4] . However, according to the phylogenetic results of coat protein genes, SCMV isolates are divided into five groups (A to E) specific to host origins [4]. Isolates of group E (SO) have been reported from sugarcane (*Saccharum officinarum*) in Vietnam and China [4] and are therefore designated as group SO. In 2015 and 2016, SCMV isolates of group SO was reported to infect *Cannas* spp*.* [5]. But so far there is no information on the complete genomic sequences of SCMV from group SO available.

## Provenance of virus material

Plant of *Canna* spp. showing symptoms of severe mosaic and dwarf were observed at incidence of 20%~ 30%. Two symptomatic leaf samples were collected in 2015 and 2016, respectively, from the parks of Ji’nan (N36°43′, E117°36′) and Tai’an (N36°10′, E117°10′), Shandong Province, China. Total RNA was extracted using the RNAprep Pure Plant Kit (Polysaccharides & Polyphenolics-rich) (Tiangen, Beijing, China). The first-strand cDNA was synthesized using Moloney MLV reverse transcriptase (Takara, Dalian, China) with primer 5’-GGTCGACTGCAGGATCCAAGCT_15_–3′ [6]. Degenerate primers was designed according to the conserved regions of potyviruses and were used to amplify different genomic parts of virus via RT-PCR [7]. To determine the genomic ends, rapid amplification of cDNA ends (RACE) was conducted using the 5’-Full RACE Kit with TAP (Takara, Dalian, China) with specific primers. An additional WORD file shows the primer sequences in more detail (see Additional file 1). DNASTAR 7.1.0 package (DNASTAR Inc., Madison, WI, USA) was used to assemble the sequences. Phylogenetic relationships were inferred by the Neighbor-Joining and Maximum Likelihood methods with 1000 bootstrap replicates using MEGA6.06.

## Sequence properties

Both of the two *Canna* samples were positive to SCMV antiserum in ELISA detection. The SCMV infecting *Canna* plants in Ji’nan and Tai’an were designated as Canna-Ji’nan and Canna-Tai’an accordingly. The complete genome of SCMV Canna-Ji’nan and Canna-Tai’an have been deposited in the GenBank database and allocated the accession numbers KY548506 and KY548507, respectively. The complete genome of both SCMV canna isolates were 9576 nucleotides (nt) in length excluding the poly (A) tails. Their 5’-UTRs and 3’-UTRs were 148 nt and 238 nt, respectively. The conserved potyboxes ‘a’ and ‘b’ in 5’-UTR [8] were identified as A_19_CAACAC_25_ and C_38_CAAGCA_44_. The first in-frame AUG_149–151_ situated in the context CGAGAUGGC was presumed to be the initiation codon of the polyprotein translation. The ORF of SCMV canna isolate was 9192 nt, encoding a polyprotein 3063 amino acids (aa). The polyprotein of SCMV is processed into ten mature functional proteins, and the nine putative protease cleavage sites were identified (Table 1) [9]. Unlike Y/A in other SCMV isolates, the cleavage site at the P1/HC-Pro junction of Canna-Ji’nan and Canna-Tai’an was Y/S, which was also found in that of *Johnsongrass mosaic virus* (JGMV). The cleavage site at VPg/NIa-Pro junction of other SCMV isolates was E/S, while that of Canna-Ji’nan and Canna-Tai’an was E/A. E/A was also found in the cleavage site at VPg/NIa-Pro junction of *Tobacco vein banding mosaic virus* (TVBMV) [6]. The highly conserved G1~2A6~ 7 (G_2 686_GAAAAAA_2 693_) motif was also identified within the P3 protein, producing PIPO with the + 2 reading frameshift.

**Table 1:** Putative cleavage sites of SCMV canna isolate and other isolates

Isolate	Canna	JN021933	AJ278405	AJ310102	AM110759
Region					
P1/HC-Pro	LDIEHY/S	LDIDHY/A	LEIEHY/A	FDIEHY/A	MEIEHY/A
HC-Pro/P3	REYIVG/G	REYIVG/G	RDYLVG/G	REYIVG/G	REYIVG/G
P3/6 K1	TGVIHE/G	TGVIHE/G	TGVIHE/G	TGVIHE/G	TGVIHE/G
6 K1/CI	TPVTHQ/S	PSVAQQ/S	PPVMQQ/S	PPVVQQ/S	PPVTQQ/S
CI/6 K2	NTVIHQ/G	NTVIHQ/G	NTVIHQ/G	NTVIHQ/G	NTVIHQ/G
6 K2/NIa-VPg	TDVLHQ/G	INVSHQ/G	TNVSHQ/G	TNVSHQ/G	TEVLHQ/G
NIa-VPg/NIa-Pro	IGVTHE/A	TGVAHE/S	TGVAHE/S	AGVAHE/S	TGVAHE/S
NIa-Pro/NIb	SSVEEQ/C	MSVEEQ/C	MIVEEQ/C	ISVEEQ/C	MSVEEQ/C
NIb/CP	EDVVHQ/A	EEVIHQ/S	DEVFHQ/A	EDVFHQ/S	EDVFHQ/S

The complete genome of SCMV Canna-Ji’nan and Canna-Tai’an shared nt identity of 98.0% and aa identity of 98.8%. At the complete genome level, Canna-Ji’nan shared the highest nt identities of 76.5% with isolates Henan (AF495510) and SX (AY569692), aa identities of 85.5% with isolates Xiangshan (AJ310103) and Yuhang (AJ310104) from China and isolate sp. (AM110759) from Spain; while Canna-Tai’an shared the highest nt identities of 76.6% with isolates Beijing (AY042184) from China and JAL-1 (GU474635) from Mexcio, aa identities of 85.6% with isolates Xiangshan (AJ310103) and Yuhang (AJ310104). At the coat protein gene level, Canna-Ji’nan and Canna-Tai’an shared nt identities of 87–91% and 87–92% with isolates from Yunnan province, China and Vietnam (FM998893-FM998896 and DQ925427-DQ925428) in GenBank, which clustered into SO group based on cp gene. The most variable gene P1 shared the lowest nt identities of 56.7–62.2% and aa identities of 51.5–55.8%, while PIPO shared the highest nt identities of 87.1–91.2% and aa identities of 85.0–92.5% among the eleven mature proteins between SCMV canna isolates and other isolates. An additional WORD file shows the nt and aa identities in more detail (see Additional file 2).

**Fig. 1:**
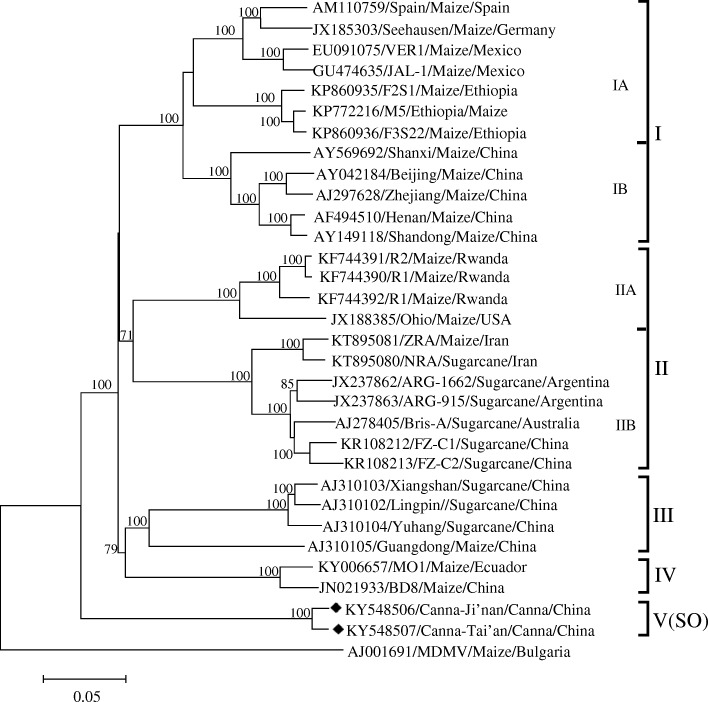
Phylogenetic tree of SCMV constructed with the complete genome sequences using the neighbor-joining method in MEGA6.06 program. The SCMV isolates were listed as accession number/isolate name/host plant/country of origin. Bootstrap analysis was applied using 1000 replicates. Only bootstrap values higher than 70% were shown. One *Maize dwarf mosaic virus* isolate (AJ001691) was used as an out-group. ◆ indicated the isolate in this study. The scale bars showed a genetic distance of 0.05

A phylogenetic tree was constructed with the complete genomic sequences of Canna-Jinan, Canna-Tai’an and other 29 SCMV isolates, using that of a MDMV isolate (AJ001691) as the out-group. The results showed that these 31 SCMV isolates were clustered to five groups. Group I contained 12 SCMV isolates from maize and was divided into two subgroups. The isolates of subgroup IA were collected from Europe, Africa and America, while those of subgroup IB were from China. Group II contained 11 isolates and was also divided into two subgroups. Subgroup IIA contained four isolates from maize, three from Africa and one from America; subgroup IIB contained six isolates from sugarcane in America and Asia, the other one from maize in Iran was actually a recombinant [10]. Group III contained four isolates from China, among which three from sugarcane and one from maize. Group IV included only two isolates from maize. BD8 was a high virulent isolate in China and had spread rapidly [4, 11]; MO1 was a isolate from Ecuador. Group V(SO) contained the Canna-Ji’nan and Canna-Tai’an. Because there was no complete genomic sequence of SO group available, SCMV was clustered into four groups in phylogenetic trees in the previous studies [4] (Fig. 1).

Canna is commonly cultivated as an important ornamental plant in the world. The canna virus disease has been increasing in the past years. The viruses reported to infect canna included *Cucumber mosaic virus* (CMV) [12, 13], *Tomato aspermy virus* (TAV) [14], *Bean yellow mosaic virus* (BYMV) [15], *Canna yellow mottle virus* (CaYMV) [12], *Canna yellow streak virus* (CaYSV) [16] and *Sugarcane mosaic virus* (SCMV) [5]. We hypothesized that the SCMV isolates of SO lineage were transmitted to *Canna* plants by aphids and experienced distinct evolution events. To our knowledge, this is first complete genomic sequences of SCMV SO group and also the first complete genomic sequences of SCMV infecting canna.

## Additional files

Additional file 1: SCMV primer sequences. (DOC 16 kb)

Additional file 2: SCMV nucleotide and amino acid sequence identities. (DOC 102 kb)

## Abbreviations

aa: amino acids
BYMV: *Bean yellow mosaic virus*
CaYMV: *Canna yellow mottle virus*
CaYSV: *Canna yellow streak virus*
CMV: *Cucumber mosaic virus*
JGMV: *Johnsongrass mosaic virus*
kDa: kilodalton
MDMV: *Maize dwarf mosaic virus*
nm: nanometer
nt: nucleotides
ORF: open reading frame
RdRp: RNA-dependent RNA polymerase
SCMV: *Sugarcane mosaic virus*
TAV: *Tomato aspermy virus*
TVBMV: *Tobacco vein banding mosaic virus*
UTR: untranslated region
