# Supplementary material for: The complete genomic sequence of Sugarcane mosaic virus from Canna spp. in China
Source: Virol J. 2018 Sep 24;15:147. doi: 10.1186/s12985-018-1058-8 (PMC6154414; doi:10.1186/s12985-018-1058-8)
Supplement: Supplementary file 1 — SCMV primer sequences. (DOC 16 kb) [file 12985_2018_1058_MOESM1_ESM.doc]

Additional file 1 Primers used for the amplification of *Sugarcane mosaic virus* canna isolate genome

| Primer name | Sequence |
| --- | --- |
| NIb-Poty | 5'-GGNAAYAAYAGYGGNCARCC-3’ |
| SCMV-R1 | 5'-ATGTATTCGAAGTCGGGATGC-3' |
| NIa-Poty | 5'-TWYTGGAADCAYTGGAT-3' |
| SCMV-R2 | 5'-TCGATGCTAGACTGTGTATG-3' |
| CI-Poty | 5'-GTNGGNTCNGGNAANTCNAC-3' |
| SCMV-R3 | 5'-AGTGTTTTGCTTCATGGTTCTTC-3' |
| HC-Pro-Poty | 5'-TGYGAYAAYCARTTNGA-3' |
| SCMV-Race-Out | 5'-GTACATACTTGGAGGTGACG-3' |
| SCMV-Race-In | 5'-CTTACACACGCTGAACTAAGAG-3' |
