# Supplementary material for: The complete genomic sequence of Sugarcane mosaic virus from Canna spp. in China
Source: Virol J. 2018 Sep 24;15:147. doi: 10.1186/s12985-018-1058-8 (PMC6154414; doi:10.1186/s12985-018-1058-8)
Supplement: Supplementary file 2 — SCMV nucleotide and amino acid sequence identities. (DOC 102 kb) [file 12985_2018_1058_MOESM2_ESM.doc]

Additional file 2 Percent nucleotide/amino acid sequence identity of the complete genome, individual ORF and untranslated region of SCMV canna isolate from Ji’nan with canna isolate from Tai’an and other 30 SCMV isolates

| Isolate | 5'-UTR | P1 | HC-Pro | P3 | 6K1 | CI | 6K2 | VPg | NIa-Pro | NIb | CP | PIPO | 3'-UTR |
| --- | --- | --- | --- | --- | --- | --- | --- | --- | --- | --- | --- | --- | --- |
| Canna-Tai'an | 97.3 | 96.4/95.7 | 98.1/99.3 | 98.3/99.1 | 98.5/100 | 97.5/98.4 | 97.5/96.2 | 97.9/99.5 | 99.0/99.6 | 97.9/99.6 | 98.2/98.7 | 100/100 | 99.6 |
| AF494510 | 66.9 | 61.7/54.5 | 79.3/92.2 | 78.1/83.6 | 74.1/79.1 | 78.6/92.8 | 71.1/75.5 | 75.3/84.7 | 77.0/88.8 | 77.4/89.8 | 76.4/78.7 | 89.6/88.8 | 79.8 |
| AJ278405 | 70.1 | 58.9/53.2 | 78.3/92.0 | 76.6/83.3 | 73.1/79.1 | 78.5/92.2 | 69.2/71.7 | 77.6/86.8 | 76.3/86.4 | 77.4/88.1 | 79.2/78.0 | 88.8/86.2 | 83.3 |
| AJ297628 | 68.2 | 61.5/54.1 | 78.3/91.1 | 78.1/83.9 | 74.6/79.1 | 78.8/92.5 | 71.1/75.5 | 75.1/84.7 | 77.4/89.3 | 77.3/89.4 | 76.9/79.4 | 89.2/88.8 | 79.8 |
| AJ310102 | 68.9 | 62.2/53.2 | 77.1/91.5 | 77.8/83.9 | 73.6/79.1 | 78.4/92.3 | 69.8/71.7 | 75.7/87.3 | 76.3/88.8 | 76.5/90.6 | 76.9/76.5 | 90.4/92.5 | 81.8 |
| AJ310103 | 68.9 | 61.7/53.6 | 77.2/91.5 | 78.2/83.9 | 74.6/79.1 | 78.1/92.8 | 70.4/71.7 | 75.7/86.8 | 78.0/89.3 | 76.8/90.8 | 77.3/78.4 | 90.0/91.2 | 82.3 |
| AJ310104 | 68.9 | 61.7/54.1 | 76.2/91.1 | 77.9/83.6 | 74.1/79.1 | 78.1/93.1 | 71.1/71.7 | 75.8/86.8 | 77.7/88.4 | 75.9/90.8 | 76.5/78.4 | 90.0/90.0 | 81.4 |
| AJ310105 | 70.3 | 59.5/50.2 | 77.7/91.3 | 78.3/83.0 | 75.1/83.6 | 79.2/92.6 | 73.0/69.8 | 76.7/88.4 | 77.4/89.3 | 77.0/89.6 | 76.3/79.0 | 88.8/88.8 | 78.1 |
| AM110759 | 66.9 | 60.2/53.2 | 78.6/91.1 | 78.3/84.4 | 74.1/79.1 | 78.4/93.1 | 72.3/77.4 | 75.8/86.2 | 74.4/89.3 | 78.0/90.8 | 75.5/77.4 | 91.2/92.5 | 79.0 |
| AY042184 | 68.9 | 60.8/54.1 | 78.8/91.1 | 77.9/84.4 | 75.1/79.1 | 78.9/92.6 | 71.1/75.5 | 75.3/85.2 | 77.0/88.4 | 77.4/89.6 | 76.8/79.0 | 89.6/88.8 | 79.4 |
| AY149118 | 67.6 | 60.8/54.9 | 79.4/92.2 | 77.7/83.0 | 74.6/79.1 | 78.8/93.3 | 71.1/75.5 | 75.5/85.2 | 77.0/88.8 | 77.2/89.8 | 76.3/79.7 | 89.6/88.8 | 79.4 |
| AY569692 | 67.6 | 61.4/53.6 | 78.3/92.0 | 78.1/84.1 | 76.6/82.1 | 79.0/92.2 | 71.7/75.5 | 76.7/85.2 | 77.1/88.8 | 77.3/89.1 | 76.8/79.4 | 89.6/88.8 | 79.4 |
| EU091075 | 66.2 | 60.5/51.5 | 78.4/91.7 | 77.7/82.4 | 73.1/80.6 | 78.8/92.5 | 70.4/73.6 | 76.2/86.2 | 74.9/89.3 | 77.8/90.6 | 77.0/77.7 | 88.3/86.2 | 85.8 |
| GU474635 | 66.9 | 60.4/51.9 | 78.9/91.3 | 78.5/83.6 | 73.1/80.6 | 79.4/92.9 | 72.3/77.4 | 75.3/86.2 | 75.2/88.8 | 77.8/90.6 | 75.7/74.7 | 88.8/87.5 | 85.0 |
| JN021933 | 66.2 | 60.9/55.4 | 78.3/92.4 | 79.8/83.3 | 73.6/80.6 | 78.9/93.1 | 73.0/69.8 | 75.5/85.2 | 75.2/87.2 | 78.0/88.3 | 75.6/76.5 | 88.8/88.8 | 78.1 |
| JX185303 | 62.9 | 59.4/53.2 | 78.5/91.5 | 78.3/84.4 | 73.6/76.1 | 78.1/92.3 | 72.3/77.4 | 76.0/86.8 | 74.9/88.0 | 77.7/90.6 | 76.5/78.4 | 91.2/91.2 | 84.0 |
| JX188385 | 70.3 | 56.7/53.6 | 76.4/91.1 | 77.7/82.7 | 72.1/77.6 | 77.1/91.8 | 74.2/73.6 | 75.3/85.2 | 75.8/88.4 | 77.5/88.1 | 76.4/76.6 | 90.8/90.0 | 79.0 |
| JX237862 | 69.4 | 59.7/55.4 | 78.0/92.0 | 76.8/83.3 | 74.1/79.1 | 78.3/92.3 | 69.8/69.8 | 77.1/87.3 | 78.1/86.4 | 78.1/89.4 | 78.7/78.9 | 87.1/85.0 | 83.7 |
| JX237863 | 71.4 | 59.7/54.9 | 77.8/91.7 | 76.8/83.6 | 72.6/79.1 | 78.2/92.3 | 68.6/69.8 | 77.1/86.2 | 77.3/86.0 | 77.5/89.6 | 79.8/77.9 | 88.3/87.5 | 81.1 |
| KF744390 | 66.4^a^ | 59.2/54.9 | 78.2/91.7 | 77.8/82.1 | 70.6/77.6 | 77.1/91.5 | 73.0/75.5 | 77.1/86.2 | 74.9/87.6 | 77.7/88.3 | 76.8/77.9 | 90.0/90.0 | 80.7^a^ |
| KF744391 | 66.2^a^ | 59.1/54.9 | 78.3/91.7 | 77.6/81.8 | 71.1/77.6 | 77.1/91.7 | 73.0/75.5 | 77.1/85.7 | 75.2/87.6 | 77.9/88.3 | 76.9/77.6 | 90.0/90.0 | 80.7^a^ |
| KF744392 | 68.3^a^ | 57.2/53.2 | 78.3/91.3 | 77.6/81.8 | 70.1/77.6 | 77.2/91.5 | 75.5/73.6 | 75.7/85.7 | 76.4/88.4 | 78.3/89.1 | 76.8/77.9 | 90.0/90.0 | a |
| KP772216 | 66.9 | 59.2/54.9 | 78.2/91.7 | 77.8/82.1 | 70.6/77.6 | 77.1/91.5 | 73.0/75.5 | 77.1/86.2 | 74.9/87.6 | 77.7/88.3 | 76.8/77.9 | 90.0/90.0 | 80.2 |
| KP860935 | 66.0 | 58.9/52.4 | 78.7/91.3 | 78.5/83.6 | 74.1/77.6 | 78.6/93.1 | 71.7/75.5 | 75.7/85.2 | 76.0/87.6 | 77.6/88.7 | 78.1/78.1 | 91.2/92.5 | 79.8 |
| KP860936 | 66.9 | 59.1/51.9 | 78.6/91.7 | 76.9/83.9 | 75.1/79.1 | 78.5/93.1 | 72.3/75.5 | 75.0/85.2 | 76.0/88.0 | 77.2/88.5 | 76.8/77.2 | 91.2/92.5 | 80.3 |
| KR108212 | 70.7 | 59.1/54.9 | 77.3/91.3 | 77.2/83.9 | 73.1/77.6 | 78.6/92.2 | 67.9/67.9 | 76.9/86.8 | 76.6/86.4 | 77.6/89.4 | 79.0/78.2 | 88.8/86.2 | 83.7 |
| KR108213 | 72.8 | 58.9/55.4 | 78.3/91.1 | 76.7/83.6 | 73.1/77.6 | 78.4/92.0 | 69.8/69.8 | 77.8/86.2 | 76.0/84.7 | 77.5/89.3 | 79.2/78.9 | 88.3/86.2 | 84.4 |
| KT895080 | 70.3 | 56.9/54.1 | 77.1/92.0 | 76.8/83.0 | 69.7/77.6 | 78.7/92.3 | 66.0/71.7 | 77.1/87.3 | 77.4/85.5 | 77.5/89.8 | 79.9/79.2 | 88.3/87.5 | 84.5 |
| KT895081 | 69.6 | 56.9/54.1 | 78.3/91.5 | 79.7/83.6 | 71.1/77.6 | 78.4/92.0 | 68.6/67.9 | 78.8/86.8 | 77.0/85.5 | 77.2/89.1 | 79.4/79.2 | 88.3/87.5 | 84.1 |
| KY006657 | 70.3 | 61.5/55.8 | 78.3/93.0 | 78.1/83.3 | 71.6/80.6 | 78.0/93.1 | 73.6/69.8 | 75.5/85.2 | 75.8/87.2 | 78.1/88.7 | 76.3/76.0 | 87.9/87.5 | 81.4 |
| NC003398 | 68.2 | 61.5/54.1 | 77.1/91.1 | 76.8/83.9 | 74.6/79.1 | 78.8/92.5 | 71.1/75.5 | 75.1/84.7 | 77.4/89.3 | 77.3/89.4 | 76.9/79.4 | 89.2/88.8 | 79.8 |

^a^ SCMV isolate KF744390-KF744392 were just nearly complete genome
